# Highly Efficient Temperature Inducible CRISPR-Cas9 Gene Targeting in *Drosophila suzukii*

**DOI:** 10.3390/ijms22136724

**Published:** 2021-06-23

**Authors:** Ying Yan, Yukino Kobayashi, Cong Huang, Bo Liu, Wanqiang Qian, Fanghao Wan, Marc F. Schetelig

**Affiliations:** 1 Department of Insect Biotechnology in Plant Protection, Institute for Insect Biotechnology, Justus-Liebig-University Giessen, Winchesterstraße 2, 35394 Giessen, Germany; Yukino.Kobayashi@ernaehrung.uni-giessen.de (Y.K.); Marc.Schetelig@agrar.uni-giessen.de (M.F.S.); 2 Shenzhen Branch, Guangdong Laboratory of Lingnan Modern Agriculture, Agricultural Genomics Institute at Shenzhen, Chinese Academy of Agricultural Sciences, Shenzhen 518120, China; huangcong16@163.com (C.H.); liubo03@caas.cn (B.L.); qianwanqiang@caas.cn (W.Q.); wanfanghao@caas.cn (F.W.); 3 Genome Analysis Laboratory of the Ministry of Agriculture and Rural Affairs, Agricultural Genomics Institute at Shenzhen, Chinese Academy of Agricultural Sciences, Shenzhen 518120, China

**Keywords:** spotted-wing drosophila, insect transgenesis, targeted mutagenesis, conditional system, pigmentation, genetic control, gene drive

## Abstract

The spotted-wing Drosophila (*Drosophila suzukii* Matsumura) is native to eastern Asia, but has become a global threat to fruit production. In recent years, CRISPR/Cas9 targeting was established in this species allowing for functional genomic and genetic control studies. Here, we report the generation and characterization of Cas9-expressing strains of *D. suzukii*. Five independent transgenic lines were generated using a *piggyBac* construct containing the *EGFP* fluorescent marker gene and the *Cas9* gene under the control of the *D. melanogaster heat shock protein 70* promoter and 3’UTR. Heat-shock (HS) treated embryos were analyzed by reverse transcriptase PCR, revealing strong heat inducibility of the transgenic *Cas9* expression. By injecting gRNA targeting *EGFP* into one selected line, 50.0% of G_0_ flies showed mosaic loss-of-fluorescence phenotype, and 45.5% of G_0_ flies produced G_1_ mutants without HS. Such somatic and germline mutagenesis rates were increased to 95.4% and 85.7%, respectively, by applying a HS. Parental flies receiving HS resulted in high inheritance of the mutation (92%) in their progeny. Additionally, targeting the endogenous gene *yellow* led to the lack of pigmentation and male lethality. We discuss the potential use of these efficient and temperature-dependent Cas9-expressing strains for the genetic studies in *D. suzukii*.

## 1. Introduction 

The Spotted Wing Drosophila, *Drosophila suzukii* (Diptera: *Drosophilidae*), is a major pest of soft-skinned fruits. It was not considered a severe problem for agriculture for decades until it arrived in Europe and North America in 2008, and its subsequent rapid spread on these continents [1,2]. *Drosophila suzukii* was recently detected on the South American and African continents [3,4], and potential invasions to Australia were predicted based on suitable environments in some regions [5]. It also causes growing damage in the native Asian area, such as China, due to climatic conditions and farming practices [6,7]. Consequently, *D. suzukii* has become a serious threat to fruit production and trade in all major continents [5,6,8]. Whole-genome assemblies of *D. suzukii* have been generated using short and long-read sequencing technologies [9,10,11], and a whole-genome scan for 22 geographic populations has been conducted to detect genetic variants associated with its invasion success [12]. These studies established the basis for functional genomic studies and genetic control strategies in this species. 

CRISPR/Cas9 systems have been a powerful genetic technology for insect mutagenesis, which can be used in general for gene targeting via non-homologous end joining (NHEJ) or transgene integration via homology-directed repair (HDR) [13,14], and specifically also in *D. suzukii* [15,16,17]. CRISPR-based genetic control strategies have then been proposed for *D. suzukii*, including gene drive that targets insect viability or fertility [18], CRISPR/Cas9 sex-distortion (CRISPR^SD^) that targets DNA sequences uniquely located on the X chromosome for biasing sex ratios [19], and precision-guided SIT (pgSIT) that simultaneously targets insect viability and fertility [20,21]. These methods would require *D. suzukii* strains that express the *Cas9* gene in the germline for heritable mutation(s). In addition, a conditional system for *Cas9* expression would be highly advantageous in those strategies to induce the sterile, lethal, or sex sorting effects, while efficiently rearing the strains in the laboratory or during mass rearing. Here, we report the generation of transgenic *D. suzukii* lines that express *Cas9* in both soma and germline. The *Cas9* gene is under control of the *D. melanogaster heat shock protein 70* gene (*Dmhsp70*) promoter [22], and its expression was compared in independent transgenic lines under different heat-shock (HS) conditions. The RNA-guided DNA cleavage efficiencies of such Cas9-expressing strains at different HS conditions were further investigated by targeting an exogenous gene, the enhanced green fluorescent protein (*EGFP*). In addition, mutagenesis of the endogenous *D. suzukii* gene *yellow* (*DsY*) was achieved, and the resulting phenotypes were verified. We discuss the possible functions of *DsY* and the potential use of the presented Cas9-expressing strains for functional genomics and genetic control studies in *D. suzukii*. 

## 2. Results

### 2.1. Generation and Characterization of D. suzukii Cas9-Expressing Lines 

**Figure 1:**
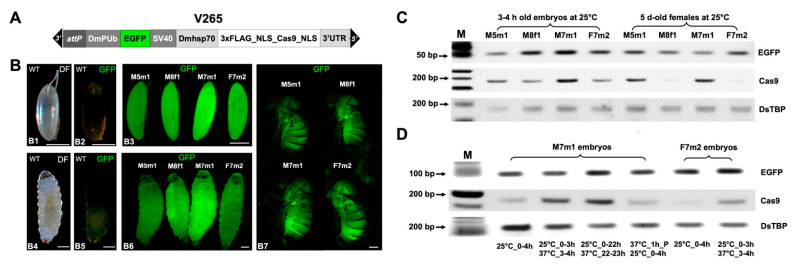
Phenotypical and molecular characterization of the *D. suzukii* Cas9-expressing lines. (**A**) Schematic map of the *piggyBac* germline transformation vector V265 (not to scale). The *enhanced green fluorescence protein* (*EGFP*) marker is regulated by *D. melanogaster polyubiquitin* promoter (*DmPUb*) and the SV40 polyA. The *Cas9* gene with a 3xFLAG protein tag and nuclear localization signals (NLS) is regulated by the *D. melanogaster heat shock protein 70* gene promoter (*Dmhsp70*) and its 3’UTR. V265 also contains an *attP* recombination site. (**B**) The dark field (DF) or enhanced green fluorescence (GFP) images of 3–4 h (h) wild type (WT) or transgenic embryos (B1–B3), WT or the transgenic third instar larvae (B4–B6), and five days (d) old transgenic females are showed. Transgenic flies were from four independent V265 homozygous lines (M5m1, M8f1, M7m1, and F2f2). Filter sets depicted in the images are described in the Method section. Scale bar: 0.2 mm for B1–B3, 0.5 mm for B4–B7. (**C**) Reverse transcriptase (RT)-PCR was used to compare RNA levels of *EGFP*, *Cas9*, and the housekeeping gene *DsTBP* from embryos (3–4 h old) and adult females (5 d old) at 25 °C for all four strains. PCR product sizes from the target genes are 79 bp for *EGFP*, 175 bp for *Cas9*, and 182 bp for *DsTBP*. (**D**) RT-PCR to compare the *Cas9* expression at different heat-shock conditions. RNA was extracted from M7m1 and F7m2 embryos. The temperature and embryonic developmental time window of treatment are depicted; Parental flies (P) were treated at 37 °C for 1 h before egg collection; M: molecular ladder.

The V265_*pBXL_attP220_PUb_EGFP_SV40_Dmhsp70_3xFLAG-NLS-Cas9-NLS_Dmhsp70-3’UTR* vector contains a codon-optimized *Cas9* under the control of the *Dmhsp70* promoter and its 3’UTR [22] (Figure 1A). The SV40 nuclear localization signal (NLS) was attached to the N and C terminus of *Cas9* for nuclear compartmentalization in cells [23]. Five independent *D. suzukii* V265 lines could be established from 77 G_0_ adults (32 males, 45 females) after *piggyBac*-mediated transformation, and four of them were bred to homozygosity (M5m1, M7m1, M8m1, F7m2). According to segregation analysis, all homozygous lines carried a single transgene on one of the autosomes and could be stably reared in the laboratory for three years. To characterize the transgene activity of different V265 lines, fluorescence images from certain developmental stages were taken as reference for *EGFP* expression (Figure 1B). Line M7m1 showed the strongest green fluorescence in embryos and larvae among the four homozygous lines. For adults, the differences in fluorescence intensity are less pronounced and flies could rather be distinguished by tissue-specific patterns. For example, M7m1 females exhibited bright fluorescence in the abdomen while M8f1 females showed bright fluorescence in the thorax (Figure 1B). 

Reverse transcriptase (RT)-PCR was then used to compare the basal and induced expression of transgenes in V265 lines on a semi-quantitative level (Figure 1C). For basal expression at 25 °C, M7m1 embryos had the highest *EGFP* and *Cas9* expression. For females collected at 25 °C, M5m1 and F7m2 lines showed higher *EGFP* expression, and M5m1 and M7m1 lines higher *Cas9* expression, when compared to those from other lines (Figure 1C). The M7m1 line having the strongest *Cas9* expression in embryos, and the line F7m2 showing relatively weak *Cas9* expression in both embryos and adults, were selected to investigate the heat inducibility of *Cas9* expression further. In case of the M7m1 line, a 1 h HS of embryos that were either 3 or 22 h old, increased the *Cas9* expression compared to that from embryos without HS (Figure 1D). However, applying HS to the parents before egg laying had no detectable impact on the *Cas9* expression level in F1 embryos. For the F7m2 line, the *Cas9* expression was almost undetectable in the embryos without HS, but largely increased after 1 h exposure at 37 °C (Figure 1D). The expression of *EGFP* and a housekeeping gene *DsTBP* was not affected by these heat shock treatments, indicating that the heat-induced *Cas9* expression was conferred by the *Dmhsp70* promoter rather than genomic position effects. 

### 2.2. Efficient and Temperature-Dependent Mutagenesis of an EGFP Transgene

**Figure 2:**
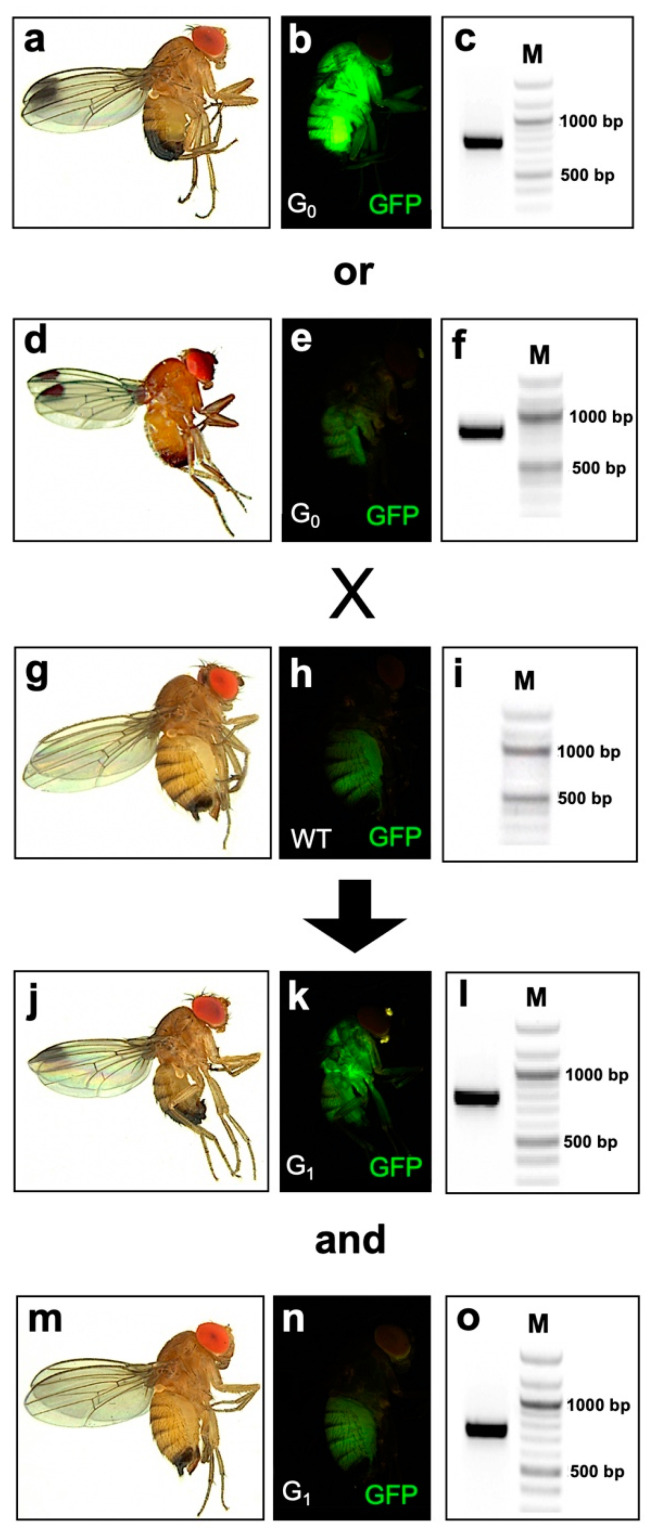
Crossing scheme of G_0_ individuals injected with gRNA targeting *EGFP* gene and molecular analysis of G_0_ and G_1_ flies. Shown are fly images in bright field (**a**,**d**,**g**,**j**,**m**) and the corresponding GFP filter (**b**,**e**,**h**,**k**,**n**) as well as the respective PCR validating the presence or absence of the *EGFP* marker gene (**c**,**f**,**i**,**l**,**o**). M: molecular ladder. After the injection of gRNA-expressing plasmid or in vitro synthesized gRNA, V265_M7m1 G_0_ individuals, which are homozygous for the *EGFP* marker gene, showed either strong whole-body fluorescence (**b**) or no fluorescence (**e**) that is similar to the phenotype (**h**) of wild type (WT). Those G_0_ were individually crossed to WT flies, and G_1_ offspring was either heterozygous for the *EGFP* (**k**) and positive in *EGFP*-specific PCR (I), or phenotypically missing the EGFP fluorescence (**n**), but still carrying the *EGFP* gene (**o**). PCR product size for *EGFP* is 782 bp. M = molecular ladder; bp = base pair.

**Figure 3:**
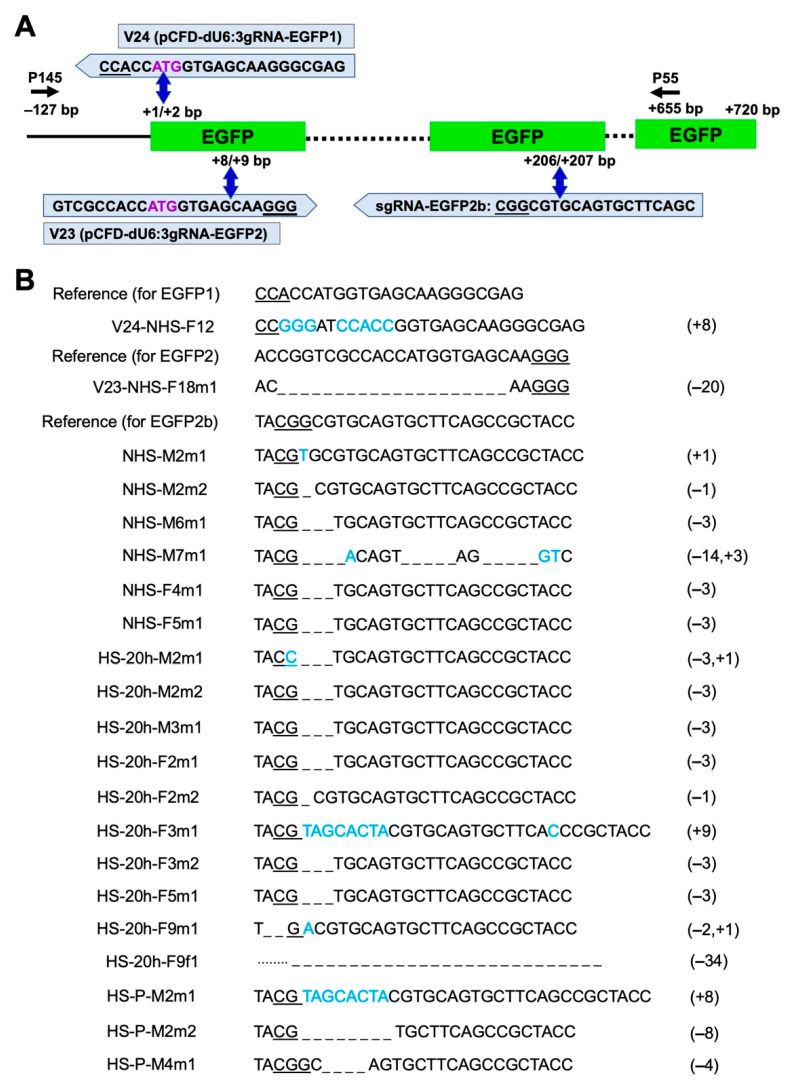
Targeted mutations in an exogenous *EGFP* gene using Cas9-expressing *D. suzukii*. (**A**) Position of gRNAs, protospacer adjacent motifs (PAM), double-strand breaks (DSB), and genotyping primers. The *EGFP* coding region in V265 construct is 720 bp (+1, +720; boxes are not to scale). PAM sequences are underlined, and predicted DSB sites are indicated by blue double-headed arrows. Plasmid V24 contains a U6:3-gRNA-EGFP1 cassette, which produces gRNA targeting the translation start codon (ATG) of *EGFP* (marked purple). Plasmid V23 contains a U6:3-gRNA-EGFP2 cassette, which produces gRNA targeting the 5’-proximal region of *EGFP*. sgRNA-EGFP2b is in vitro synthesized gRNA, and the target site is at +206/207 bp of *EGFP*. Relative to the *EGFP* sequence, gRNA-EGFP2 is sense-oriented while gRNA-EGFP1 and gRNA-EGFP2b are anti-sense-oriented. The black arrows show the PCR primers P145 (located at −127 bp relative to *EGFP*) and P55 (located at +655 bp of *EGFP*), which were used to detect the mutations. (**B**) Sequences of mutant *EGFP* alleles identified in G1 individuals compared to the *EGFP* reference sequence. Mutants from certain families derived from different injections are shown on the left. V23 and V24 were injected under no heat-shock (NHS) conditions. The sgRNA-EGFP2b-injected embryos were subject to NHS or heat shock 20 h after the injection (HS-20h), or the embryos were collected from parents that subject to heat shock (HS-P). The numbers of deleted (_) or inserted (in blue) nucleotides are indicated in the brackets on the right of each sequence.

First, Cas9-expressing line M7m1 was injected individually with two gRNA-expressing plasmids, V24_dU6:3-EGFP1 and V23_dU6:3-EGFP2, to target *EGFP* and evaluate the DNA cleavage efficiency of the transgenic Cas9 component. The injected G_0_ flies were individually backcrossed to wild type (WT) flies, and G_0_ and G_1_ flies were screened for loss of green fluorescence and genotyped for mutation events (Figure 2 and Figure 3). Both gRNA plasmids targeted around the start codon sequence of *EGFP* and were injected into M7m1 embryos without applying a HS (Figure 3A). The injection of plasmid V24 led to one mosaic individual out of 23 G_0_ adults (4.3% mosaic rate) with a verified mutation in the targeted region, but no G1 mutants from a total of 14 fertile crosses (Figure 3B; Table 1). The injection of plasmid V23 also generated one mosaic individual out of 36 G_0_ flies (2.7% mosaic rate), and G_1_ mutants were screened from one out of 33 fertile crosses (3.0% founder rate) (Figure 3B; Table 1). 

Second, in vitro synthesized gRNA-EGFP2b targeting the chromophore region of *EGFP* [24] was injected following different HS conditions. Without HS, 50% of the G_0_ adults injected with gRNA-EGFP2b completely lost the green fluorescence, indicating efficient somatic mutations of *EGFP* (Figure 2; Table 1). Out of eleven fertile crosses, five produced G_1_ mutants and the inheritance from G_0_ to G_1_ in different crosses ranged within 9.1–98.8% (Table S2). Applying a HS 1 h after injection resulted in low larval hatch (2.6%) and adult survival rates (0.5%) significantly lower than those from injections without HS (Table 1; *p* < 0.05, Z test). This suggests that the temporarily elevated temperature is harmful to young embryos and such treatment only generated two fertile G_0_ adults out of 557 injected eggs, and no G_1_ mutants were found (Table 1). Applying a HS 20 h after injection showed relatively high larval hatch (18%) and adult survival rates (4.3%), indicating that old embryos are more resistant to HS and survive better than young embryos. Under such conditions, 21 out of 22 G_0_ adults lost the green fluorescence, and six out of seven fertile crosses produced G_1_ mutants, resulting in 95.4% mosaic and 85.7% founder rates, which were almost twice as high compared to injections without HS (Table 1). Notably, four G_0_ crosses produced only flies without fluorescence (Table S2), suggesting biallelic targeting in every germ cell. Applying HS to parental flies prior to egg collection and injection led to 40.9% mosaic and 20.0% founder rates (Table 1). Importantly, elevated mutation inheritance was observed from parental HS treatment (92.0%), which was significantly higher than without HS (*p* < 0.001, z = 14.207, Z test), suggesting that the maternal deposition of Cas9 plays an important role in promoting mutation inheritance. At least one mutation event was confirmed in the targeted sequence from each mutant-positive cross (Figure 3B). In addition, off-target analysis for gRNAs targeting *EGFP* showed high specificity scores (99–100%) for the selected gRNAs and no off-target sites in any CDS in the *D. suzukii* genome (Figure S1).

### 2.3. Disruption of the Endogenous Yellow Gene Causes Loss of Pigmentation and Male Lethality

**Figure 4:**
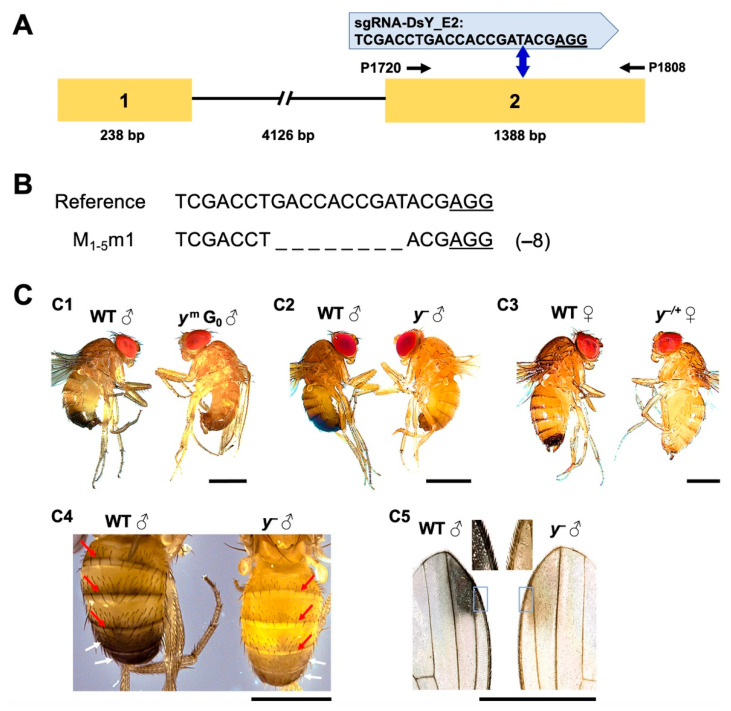
Targeted mutation in an endogenous *yellow* gene (*DsY*) using Cas9-expressing *D. suzukii*. (**A**) Schematic of *DsY* genomic organization with exons as numbered yellow boxes and intron as lines (not to scale). The PAM sequence is underlined. The predicted DSB site is indicated by blue double-headed arrows. Genotyping primers are indicated as black arrows. (**B**) Alignment of a *DsY* mutant allele isolated from *DsY^−^* flies compared to the WT (reference) sequence. The origin is shown on the left, and the numbers of deleted nucleotides (_) indicated in the brackets on the right. (**C**) Comparison between the WT (left position in each image) and *DsY^−^* mutant (right position in each image) phenotypes. Nearly whole-body mosaic yellow phenotype (*DsY^m^*) was observed in G_0_ flies (**C1**), and male mutants with the whole-body yellow phenotype (*DsY^−^*) were obtained at G_1_ (**C2**). Female heterozygous mutants (*DsY^−/+^*) were also obtained at G_1_, which showed a more yellowish pigmentation compared to WT females (**C3**). The tan pigments present in midline stripes (indicated by red arrows) and abdominal sixth and seventh segments (indicated by white arrows) from *DsY^−^* males suggest the Yellow protein was absent throughout the abdominal tergite (**C4**). WT flies are a uniform color in the wing background, spot area, and trichomes, while these structures become tan and yellowish in the *DsY^−^* males (**C5**). Higher resolution views of the regions were boxed. Scale bar: 1 mm.

To prove the functional potential of the CRISPR strains against endogenous genes, the *yellow* gene of *D. suzukii* was targeted. First, the open reading frame of *DsY* from the WT-USA strain was verified to be 1626 bp in length, encoding a protein of 541 amino acids. Sequence alignment with the alleles from two reference genomes identified 39 SNPs in the isolated *DsY* sequence, which led to one amino acid difference (519 R > G; Figure S2). The Yellow proteins are highly conserved (91.8–99.5% identity) and the Major Royal Jelly Protein domain (MRJP: PF03022) can be identified in all analyzed *Drosophila* species [25] (Figure S3). Phylogenetic analysis of 44 insect species suggested that DsYellow clusters with its ortholog in *D. biarmipes* (Figure S4). sgRNA-DsY E2 was then designed to target the second exon in *DsY,* and off-target analysis suggested it has a 100% specificity score and no off-target sites in the genome (Figure 4A and Figure S1). Due to the low mutagenesis efficiencies from plasmids targeting *EGFP* (Table 1), sgRNA-DsY_E2 was in vitro synthesized and injected into WT embryos. The nearly whole-body mosaic yellow phenotype in the G_0_ flies revealed efficient somatic mutations in the *DsY* loci (Figure 4C1). *DsY* is X-linked. Thus, hemizygous males (*DsY^−^)* and heterozygous females (*Ds^−/+^)* were obtained in G_1_ flies carrying the mutation (Table S3; Figure 4C2,C3). The characteristic loss of melanin coloring in the abdominal tergites and the abdominal sixth and seventh segments from the *DsY^−^* males (Figure 4C4) resembled the typical loss of pigmentation phenotype compared to *D. melanogaster yellow* mutants (*Dm^−^*) [26]. In addition, the beige coloring in the wing spot areas and trichomes of the *DsY^−^* males (Figure 4C5) was comparable to *D. biarmipes yellow* mutants [27,28]. Surprisingly, none of the *DsY^−^* G_1_ males survived more than two days after emergence, while most *DsY^−/+^* females survived for at least 10 days (Table S4). Therefore, *DsY^−/+^* females, which showed moderate yellowish pigmentation (Figure 4C3), were crossed to WT males in each generation for stock maintenance. To verify if the lethality is linked to the generated *DsY* mutation, *DsY^−^* males from multiple generations were genotyped, and the same mutation event was confirmed (Figure 4B). The *DsY^−^* males from consecutive 20 generations were used for the lethality tests, and the majority (66–93%) of them died within one day, and all died within two days after emergence (Table S4). 

## 3. Discussion

The development of Cas9 expression systems for gene editing in a pest species like *D. suzukii* is crucial to its efficient implementation in the laboratory or future field scenarios. Depending on the envisioned use and applications, it might be important to express the Cas9 in certain tissues, developmental time windows, or via induction from external stimuli. In this study, we generated the first Cas9 expression lines in *D. suzukii* with the option of a strong Cas9 induction via heat shock. Such a system has the flexibility to increase expression specifically at different developmental stages based on the needs of each study. The heat-inducible *D. suzukii* Cas9-expressing lines generated and validated in this study contains a *Dmhsp70-Cas9* gene cassette that was initially generated by linking an NLS to both the N and the C terminus of *Cas9* to improve localization of Cas9 to the nucleus [23] and flanked by the *Dmhsp70* promoter and 3’UTR regions [22]. The transient expression of this *Dmhsp70-Cas9* cassette targeting *yellow* of *D. melanogaster* (*DmY*) led to mutations in the soma and the germline [22] and a combination of *Dmhsp70-Cas9* plus U6:2-gRNA cassette demonstrated efficient gene editing in *D. melanogaster* (founder rates ranged from 6% to 89% depending on the targets) [29]. In addition to the use of transient sources of Cas9, multiple transgenic Cas9-expressing lines were generated in *D. melanogaster* in which the *Cas9* was regulated by either germline-specific promoters such as *nanos* and *vasa* [30,31,32,33] or ubiquitous promoters, such as *actin5C* and *Ubiquitin 63E* [34,35]. All these Cas9-expressing plasmids or insect strains greatly facilitate the mutagenesis studies in *D. melanogaster*. The transfer of the system to *D. suzukii* to express *Cas9* and validate targeting of the *EGFP* and *yellow* genes in transgenic *D. suzukii* in our study demonstrates the activity of *D. melanogaster hsp70* regulatory elements in this species. The efficiency could possibly be further improved by using endogenous elements from *D. suzukii* that have been isolated in earlier studies like the *hsp70* and *U6* promoters or NLS elements from the sex determination gene *transformer* from *D. suzukii* [17,36], but with an editing efficiency in transgenic, heat-shocked flies of up to 85.7% this seems not to be crucial. 

CRISPR-mediated gene editing using transient Cas9 expression has been achieved in *D. suzukii* by targeting a pigmentation gene *white* and a sex determination gene *sex lethal* (*Sxl*) [15], generating *white* mutant strains [37,38], creating a temperature-sensitive point mutation in the sex determination gene *transformer-2* [16], knocking out the *odorant receptor co-receptor* (*Orco*) gene [39], or knocking in fluorescent genes [17]. Among these reports, the highest reported germline transformation rate via NHEJ was 20%, which was achieved by injecting purified Cas9 protein and a synthesized gRNA targeting the *white* gene [38]. The highest HDR rate reported so far was 15.5% which was achieved by injecting *Dshsp70-Cas9* and *DsU6c-gRNA* plasmids together with a knock-in template [17]. Using the transgenic Cas9-expressing line M7m1 and synthesized gRNA targeting *EGFP*, our NHEJ rate was 45.5% without HS, more than doubling the NHEJ rate (20%) when providing Cas9 transiently [38]. This was considerably increased under HS conditions with higher mosaic (95.4%) and NHEJ rates (85.7%; Table 1), confirming the increased mutagenesis efficiency after heat-shock in the Cas9-expressing strains. 

Targeting the endogenous *DsY* gene in our transgenic Cas9 expressing line exposed a difference to *DmY* mutants. The discovered lethal phenotype in the *DsY* mutants was not reported in *yellow* mutants from other *Drosophila* species [22,27,34]. *yellow* is a pleiotropic gene required for black pigmentation, mating behavior, and potentially other traits in *Drosophila* [25,40]. Yellow mediates the production of black melanin, which is formed from the catecholamines L-3,4-dihydroxyphenylalanine (dopa) and 3,4-dihydroxyphenylethylamine (dopamine), and this synthesis pathway affects both cuticle secretion and sclerotization, which are vital for insect growth, development, and survival [40,41]. Several upstream genes in the pathway are essential for larval or adult development [42,43]. For example, the multi-ligand endocytic receptor Megalin (Mgl) controls Yellow protein levels, and its loss-of-function mutation is lethal for *D. melanogaster* [41]. In addition, silencing of a DOPA decarboxylase gene in *Rhodnius prolixus* (*RpAadc-2*) reduces the nymph survival due to the lack of cuticle pigmentation and the inability of feeding [44]. Meanwhile, *yellow* is a downstream gene of the *fruitless* (*fru*) gene, which is a member of the *Drosophila* somatic sex determination pathway, and both *fru* and *yellow* mutants show abnormal male courtship behavior and less mating success compared to wild type *D. melanogaster* [45,46]. Yellow is associated with FRU in the larval brain, and downregulation of Yellow in the central nervous system (CNS) was linked to the misregulation of mating behavior [45]. Therefore, it could be speculated that the lack of cuticle pigmentation or deficiency of CNS development was more severe in *DsY* mutants than in *DmY* mutants and might have led to the lethality of *D. suzukii* flies. 

We previously characterized the pigmentation gene *white* in *D. suzukii,* and its loss-of-function mutation (*Dsw^−^*) caused more severe phenotypes compared to those in *D. melanogaster* (*Dmw^−^*) [38]. *white* is known for its role in eye pigmentation and mating behavior, and *Dmw^−^* males showed CNS deficiency and reduced dopamine levels and mating success [47,48]. On the other hand, *Dsw^−^* males were sterile due to the lack of male courtship and copulation [38]. Meanwhile, both *white* and *yellow* are X-linked and show recessive phenotypes in *D. melanogaster.* Females carrying heterozygous mutations of *white* or *yellow* have similar pigmentation as WT females (red eyes or pigmented body) due to the dosage compensation on the X chromosome [49,50,51,52]. However, the *D. suzukii* females, which are heterozygous for *Dsw^−^* or *DsY^−^* showed brown eye or yellowish body phenotypes, respectively, that are distinguishable from WT (Figure 4 C3) [15,38]. This suggests that the X chromosome dosage compensation for these gene loci in *D. suzukii* is not as strong as in *D. melanogaster* or that the gene function of both genes has changed during the evolution of *D. melanogaster* and *D. suzukii* homologs. Because *Dsw^−^* males are sterile and *DsY^−^* males are short-lived, the visible phenotypes in *Dsw^−^* and *DsY^−^* females allowed us to identify and maintain the mutant strains by crossing them to WT males. In addition, the abnormal mating behaviors in the *DmY^−^* and *Dsw^−^* males were linked to the unpigmented sex combs and testis, respectively [38,53]. Therefore, a spatial and temporal examination of the pigmentation in *DsY^−^* males may help identify mechanical differences in the anatomy responsible for the observed lethality. 

CRISPR/Cas9 is a versatile and efficient tool for functional genomics studies in insects, however, the generated mutations could be quickly lost if they are sterile or lethal. A tissue-specific CRISPR using the UAS/Gal4 system has been developed in *D. melanogaster,* which restricts mutagenesis to certain tissues or cells, thus rescuing individuals that carry lethal mutations [54,55]. Alternatively, other conditional expression systems such as those regulated by antibiotics, non-antibiotic molecules, or external stimuli could be considered for the control of Cas9 expression [56]. Our work here verified that the Cas9 expression in the *Dmhsp70-Cas9* line is heat-inducible, and its basal expression level is associated with the genomic position of the transgene. Therefore, it should be possible to select a *Dmhsp70-Cas9* line with a minimal basal expression and a reasonable heat-inducible expression of Cas9 (such as line F7m2 in this study), which would be suitable to study sterile or lethal mutations. Such *Dmhsp70-Cas9* lines carrying gene mutation(s) could be sustained at permissive temperature, and sterile or lethal phenotypes could be screened and studied by switching to restrictive temperature. The same principle can also be considered for CRISPR-based genetic control strategies such as gene drive, CRISPR^SD,^ and pgSIT [18,21,57]. Here, the engineered strains could be efficiently maintained by suppressing the sterile, lethal, or sex-biasing effect at permissive temperatures. In addition, the HS inducible *D. suzukii* Cas9-expressing strains could provide safeguarding methods for gene drive research since they can be used for both synthetic target drive and split drive [58,59]. Specifically, these lines contain the synthetic target *EGFP* that does not exist in wild populations and provide a genomic source of Cas9. Therefore, only an unlinked gRNA construct is needed for completing a functional but split gene drive system. Such strategies can efficiently prevent unintended gene propagation even if the engineered organisms would escape [58,59]. Depending on the strategy and the specific gene target(s), further tests would be needed to evaluate the general fitness, overall competitiveness, and mutagenesis efficacy of such strains in response to temperature fluctuations in the lab, mass rearing, and later on in contained semi-field conditions.

## 4. Methods and Materials

### 4.1. Insect Rearing and Germline Transformation

The WT USA strain and transgenic *D. suzukii* lines were maintained at 25 °C and 60% humidity with a 12-h photoperiod. The germline transformation was carried out as previously reported [36]. Briefly, eggs were collected from WT-USA strain over a 30 min period on a grape juice agar plate (1% agar, water:grape juice ratio 7:3), desiccated for 10 min, and overlaid with halocarbon oil 700 (Sigma-Aldrich, St. Louis, MO, USA). A mixture of the *piggyBac* donor construct (700 ng/µL) and the phsp-pBac transposase (300 ng/µL) helper was injected into wild-type embryos. Surviving G_0_ adults were backcrossed to WT males or virgin females with putative G_1_ transformant progeny selected by EGFP fluorescence. Segregation tests were conducted by outcrossing the transformants to WT flies. Independent homozygous strains were established by screening the fluorescence intensity at the third-instar larval stage for homozygous individuals. 

### 4.2. Plasmid Construction 

To generate the *piggyBac* germline transformation vector V92_ *pBXL_attP220_PUb_EGFP_SV40*, an *attP* fragment was amplified from #1425*_pBXL_attP220_PUb_DsRed.T3_SV40* [60] with primers P212 and P213 (Table S1) and cloned into #1419*_pBXL_PUb_EGFP_SV40* [60] using the restriction enzyme Bsp119I. The *Dmhsp70_3xFLAG-NLS-Cas9-NLS_Dmhsp70-3’UTR* cassette was excised from *pBS-Dmhsp70_3xFLAG-NLS-Cas9-NLS_Dmhsp70-3’UTR* (Addgene plasmid #46294; http://n2t.net/addgene:46294, accessed on 4 January 2021) and inserted into the SacII cut V92 vector, to obtain the V265_*pBXL_attP220_PUb_EGFP_SV40_Dmhsp70_3xFLAG-NLS-Cas9-NLS_Dmhsp70-3’UTR*. V265 was sequenced using several primers (MFS10, MFS17, and P109) to confirm the integrity of the *Cas9* cassette. To generate plasmid V23_*pCFD3-dU6:3gRNA_EGFP1* and V24_*pCFD3-dU6:3gRNA_EGFP2*, DNA oligonucleotides for the selected DNA sequences were synthesized with 5’-phosphorylation, annealed and ligated to pCFD3-dU6:3gRNA (Addgene plasmid # 49410; http://n2t.net/addgene:49410, accessed on 4 January 2021) that had been digested with BbsI. 

### 4.3. Heat-Shock (HS) Treatments and Reverse Transcriptase (RT) PCR

To compare the basal gene expressions from different transgenic strains, eggs were collected from homozygous lines over one hour time windows (h) on grape juice agar plates and left on the lab bench (25 °C and 40–50% humidity) for three hours before immersion in liquid nitrogen. Newly emerged females were separated from males and aged until five days (d) old before sampling. To compare gene expressions from different HS treatments, eggs were collected from homozygous lines for 1 or 3 h at 25 °C, then immediately subject them to one of the following conditions before sampling: 1 h on the lab bench at 25 °C, or 1 h in the HS incubator (conditions: 37 ± 0.2 °C and 40–50% humidity (Binder BD23, Binder GmbH, Tuttlingen, Germany), or 20 h in the insect rearing incubator (25 °C and 60% humidity; Binder KBWF 720, Binder GmbH, Tuttlingen, Germany) followed by 1 h in the HS incubator. In addition, 5–7 d old adult flies from the transgenic line were placed in the HS incubator for 1 h and immediately used for egg collection for 1 h and eggs were aged at 25 °C for 3 h before sampling. Total RNA was isolated from the sampled eggs or adults using the ZR Tissue and Insect RNA MicroPrep kit (Zymo Research) and 1 μg of RNA was transcribed into cDNA using the iScript cDNA Synthesis Kit (Bio-Rad, Hercules, CA, USA). Each 25-μL reaction mix comprised of 0.2 μL Platinum Taq DNA polymerase (Invitrogen, Darmstadt, Germany), 2.5 µL 10× PCR buffer, 0.75 µL 50 mM MgCl_2_, 1.0 µL 10 mM dNTP mix, 1.0 µL of each primer (0.4 µM; Table S1), and 1 μL (for *EGFP*) or 2 μL (for Cas9 or *TATA-binding protein*, *TBP*) cDNA. The PCR reactions were subject to the following thermal cycling parameters: 94 °C for 2 min, 35 cycles of (94 °C for 30 s, 55 °C for *TBP* (or 65 °C for Cas9) for 30 s, 72 °C for 30 s), or 33 cycles of (94 °C for 30 s, 60 °C for *EGFP* for 30 s, 72 °C for 30 s), and final extension for 5 min at 72 °C. 

### 4.4. Gene Sequence Isolation and Analysis

The primers (Table S1) were designed based on the *yellow* gene sequence (DS10_00005318) from a *D. suzukii* reference genome [9]. The cDNA was prepared from WT-USA adult flies and the PCR reaction was assembled using the Platinum Taq polymerase (Invitrogen) as described above. An initial denaturation step at 94 °C for 2 min was followed by 35 cycles of denaturation at 94 °C for 30 s, annealing at 58 °C for 30 s and extension at 72 °C for 35 s, a final extension at 72 °C for 5 min, and a 4 °C hold. The PCR products were separated by 1% agarose gel electrophoresis and extracted from the gels using the QIAquick Gel Extraction Kit (Qiagen, Hilden, Germany) before cloning into the pCR4-TOPO vector. The presence of inserts was confirmed by restriction digestion with EcoRI and sequencing using M13 primers. Sequence translation and alignment were performed using the Geneious Prime software. Multiple sequence alignment and phylogenetic analysis were performed using MAFFT v7 (http://mafft.cbcr.jp/alignment/server/ (accessed on 4 January 2021) and MEGA6. 

### 4.5. gRNA Design, Off-Target Analysis and Injections

The design and assessment of gRNA targets and their off-targets were performed based on the *D. suzukii* reference transcripts OGS1.0 (Dsuzuki_OGS10_transcripts.fa.gz) [9] using the Geneious 11 software and a CRISPR plugin [61,62]. sgRNA-EGFP2b and sgRNA-DsY_E2 were in vitro synthesized using the HiScribe T7 High Yield RNA Synthesis Kit (NEB) as previously described [24,38]. The injection mix was prepared with either 200 ng μL^−1^ synthesized gRNA or 500 ng μL^−1^ plasmid (V23 or V24) prepared using the NucleoBond Xtra Maxi EF kit (Macherey Nagel, Düren, Germany). The injection was carried out as described earlier except that transgenic homozygous line M7m1 was used for egg collection. One of the following HS conditions was applied: (1) no HS as the parental flies and injected embryos were kept at 25 °C (BINDER, KBWF 720); (2) 1 or 20 h after injection the embryos were treated with 37 °C (BINDER, BD23) for 1 h then put back to 25 °C; (3) parental flies were treated with 37 °C for 1 h prior to the start of egg collection, and the injected embryos were kept at 25 °C. G_0_ adults were individually or group crossed to WT individuals and reared for two weeks by transferring flies to fresh food vials every 2 d at 25 °C. 

### 4.6. Crosses, Stock Keeping and Lethality Tests

For injections targeting *EGFP*, the G_0_ adults were individually crossed to WT males or virgin females, and the flies were transferred to a fresh vial by flipping every 2 d for a total of 2 weeks (eight vials in total). The G_1_ flies were collected from these vials and scored for the loss of green fluorescence. The differences in G_0_ larval hatch, adult survival, mosaic and founder rates, and mutation inheritance rate in G_1_ between different injections were analyzed by Z-tests in SigmaPlot v14 (Systat Software). For injections targeting *DsY*, G_0_ adults were group crossed to WT flies and G_1_ flies were screened for the loss of pigmentation. *DsY^−/+^* females were crossed with WT males in each generation for population maintenance. From G_1_ to G_20,_ seven or fifteen newly emerged *DsY^−^* males (<4 h) were collected in each generation and placed in a food vial. The flies were transferred to a fresh vial every 5 d and counted every day until day 10 or all flies were dead. WT males or *DsY^−/+^* females from G_1_ and G_20_ were tested in the same way.

### 4.7. Imaging and Genotyping

Flies and tissues were imaged using a Leica M205FC stereomicroscope (Leica Microsystems) with dark field, bright field, and the GFP-LP filter (excitation = 425/460 nm, emission = 480 nm). To genotype mutation events, genomic DNA was extracted from individual flies using the ZR Tissue & Insect DNA Miniprep kit (Zymo Research, Freiburg, Germany). The targeted sequences were amplified by PCR using primer pairs listed in Table S1 and the DreamTaq polymerase (Life Technologies) according to the manufacturer’s protocol. The amplification program started with a denaturation step at 95 °C for 3 min, followed by 35 cycles of 95 °C for 30 s, 58 °C (*EGFP*) or 63 °C (*DsY*) for 30 s, and 72 °C for 1 min, then a final extension step at 72 °C for 5 min. The 782-bp PCR product spanning the target sites in *EGFP* or 437-bp PCR product spanning the target site in *DsY* were analyzed by agarose gel electrophoresis. DNA was purified using the DNA Clean & Concentrator kit (Zymo Research) and sequenced using either primer P145 for *EGFP* or P1720 for *DsY*.

## Figures and Tables

**Table 1:** Mutagenesis efficiencies targeting *EGFP* using the Cas9-expressing line M7m1 and different heat-shock conditions.

Injection Mix ^a^ (ng/µL)	Heat-Shock (HS) Treatment ^b^	Injected Embryos	Hatched Larvae ^c^	G_0_ Adult Survivor ^c^	Mosaic G_0_ Adults ^d^	G_0_ Founders ^e^	Inheritance from G_0_ to G_1_^f^
V23 plasmid (500)	No HS	697	16.4% (114/697)	5.2% (36/697)	2.7% (1/36)	3.0% (1/33)	1.0%
V24 plasmid (500)	No HS	598	16.4% (98/598)	3.9% (23/598)	4.3% (1/23)	0.0% (0/14)	0.0%
EGFP-2b (200)	No HS	546	8.1% (44/546)	2.6% (14/546)	50.0% (7/14)	45.5% (5/11)	39.4% (9.1–98.8%)
EGFP-2b (200)	HS (after 1 h)	557	2.6% (12/557)	0.5% (3/557)	33.3% (1/3)	0.0% (0/2)	0.0%
EGFP-2b (200)	HS (after 20 h)	516	18.0% (93/516)	4.3% (22/516)	95.4% (21/22)	85.7% (6/7)	78.3% (3.6–100%)
EGFP-2b (200)	HS (parents)	509	10.0% (51/509)	4.3% (22/509)	40.9% (9/22)	13.3% (2/15)	92.0% (89.0–95.0%)

^a^ The injection mix contains either DNA plasmid expressing gRNA or in vitro synthesized gRNA. V23 plasmid uses the DmU6:3 promoter to regulate gRNA that targets the 5’ coding region sequence (CDS) of *EGFP* (PAM site is 8 bp downstream of ATG). V24 plasmid uses DmU6:3 promoter to regulate gRNA targeting the translation starting code of *EGFP* (PAM site is 2 bp upstream of ATG). EGFP-2b [24] is the synthesized gRNA that targets the chromophore region of *EGFP* (PAM site is 197 bp downstream of the ATG). ^b^ “No heat-shock (HS)” indicates that parental flies and embryos were kept at room temperature (25 °C). “HS (after 1 h)” or “HS (after 20 h)” indicates 1 or 20 h after injection the embryos were treated with 37 °C for 1 h then put back to 25 °C. “HS (parents)” indicates the embryos were collected from parents treated with 37 °C for 1 h prior to the egg collection. ^c^ Percentage relative to the number of embryos. ^d^ Percentage relative to the number of G_0_ adults. ^e^ Percentage of G_0_ flies that transmitted Cas9-derived mutant alleles to the G_1_ generation (founders) relative to the number of fertile G_0_. ^f^ Average proportion of G1 flies that inherited the Cas9-derived mutant alleles obtained in a two-week period (inheritance). The range in brackets shows the percentage of mutant flies produced from each individual positive cross (details are shown in Table S2).

## Data Availability

All data generated or analyzed during this study are included in this published article (and its supplementary information files). The GenBank accession numbers are as follows: pBXL_attP220_PUb_EGFP_SV40_Dmhsp70_3xFLAG-NLS-Cas9-NLS_Dmhsp70-3’UTR (V265): MN735456; DsY mRNA: MZ066636.

## Supplementary Materials

The following are available online at https://www.mdpi.com/article/10.3390/ijms22136724/s1.

## References

[1] Walsh D.B., Bolda M.P., Goodhue R.E., Dreves A.J., Lee J., Bruck D.J., Walton V.M., O’Neal S.D., Zalom F.G. (2011). *Drosophila suzukii* (Diptera: *Drosophilidae*): Invasive pest of ripening soft fruit expanding its geographic range and damage potential. J. Integr. Pest. Manag..

[2] Cini A., Ioriatti C., Anforna G. (2012). A review of the invasion of *Drosophila suzukii* in Europe and a draft research agenda for integrated pest management. Bull. Insectol..

[3] Deprá M., Poppe J.L., Schmitz H.J., De Toni D.C., Valente V.L. (2014). The first records of the invasive pest *Drosophila suzukii* in the South American continent. J. Pest Sci..

[4] Kwadha C.A., Okwaro L.A., Kleman I., Rehermann G., Revadi S., Ndlela S., Khamis F.M., Nderitu P.W., Kasina M., George M.K. (2021). Detection of the spotted wing drosophila, *Drosophila suzukii*, in continental sub-Saharan Africa. J. Pest Sci..

[5] Maino J.L., Schouten R., Umina P. (2021). Predicting the global invasion of *Drosophila suzukii* to improve Australian biosecurity preparedness. J. Appl. Ecol..

[6] Asplen M.K., Anfora G., Biondi A., Choi D.S., Chu D., Daane K.M., Gibert P., Gutierrez A.P., Hoelmer K.A., Hutchison W.D. (2015). Invasion biology of spotted wing *Drosophila* (*Drosophila suzukii*): A global perspective and future priorities. J. Pest Sci..

[7] Rogers M.A., Burkness E.C., Hutchison W.D. (2016). Current SWD IPM tactics and their practical implementation in fruit crops across different regions around the world. J. Pest Sci..

[8] Dos Santos L.A., Mendes M.F., Kruger A.P., Blauth M.L., Gottschalk M.S., Garcia F.R. (2017). Global potential distribution of *Drosophila suzukii* (Diptera, *Drosophilidae*). PLoS ONE.

[9] Chiu J.C., Jiang X., Zhao L., Hamm C.A., Cridland J.M., Saelao P., Hamby K.A., Lee E.K., Kwok R.S., Zhang G. (2013). Genome of *Drosophila suzukii*, the spotted wing drosophila. G3.

[10] Ometto L., Cestaro A., Ramasamy S., Grassi A., Revadi S., Siozios S., Moretto M., Fontana P., Varotto C., Pisani D. (2013). Linking genomics and ecology to investigate the complex evolution of an invasive *Drosophila* pest. Genome Biol. Evol..

[11] Paris M., Boyer R., Jaenichen R., Wolf J., Karageorgi M., Green J., Cagnon M., Parinello H., Estoup A., Gautier M. (2020). Near-chromosome level genome assembly of the fruit pest *Drosophila suzukii* using long-read sequencing. Sci. Rep..

[12] Olazcuaga L., Loiseau A., Parrinello H., Paris M., Fraimout A., Guedot C., Diepenbrock L.M., Kenis M., Zhang J.P., Chen X. (2020). A whole-genome scan for association with invasion success in the fruit fly *Drosophila suzukii* using contrasts of allele frequencies corrected for population structure. Mol. Biol. Evol..

[13] Sun D., Guo Z.J., Liu Y., Zhang Y.J. (2017). Progress and Prospects of CRISPR/Cas Systems in Insects and Other Arthropods. Front. Physiol..

[14] Taning C.N.T., Van Eynde B., Yu N., Ma S.Y., Smagghe G. (2017). CRISPR/Cas9 in insects: Applications, best practices and biosafety concerns. J. Insect Physiol..

[15] Li F., Scott M.J. (2016). CRISPR/Cas9-mediated mutagenesis of the *white* and *Sex lethal* loci in the invasive pest, *Drosophila suzukii*. Biochem. Biophys. Res. Commun..

[16] Li J., Handler A.M. (2017). Temperature-dependent sex-reversal by a *transformer-2* gene-edited mutation in the spotted wing drosophila, *Drosophila suzukii*. Sci. Rep..

[17] Ahmed H.M.M., Hildebrand L., Wimmer E.A. (2019). Improvement and use of CRISPR/Cas9 to engineer a sperm-marking strain for the invasive fruit pest *Drosophila suzukii*. BMC Biotechnol..

[18] Oberhofer G., Ivy T., Hay B.A. (2018). Behavior of homing endonuclease gene drives targeting genes required for viability or female fertility with multiplexed guide RNAs. Proc. Natl. Acad. Sci. USA.

[19] Tsoumani K.T., Meccariello A., Mathiopoulos K.D., Papathanos P.A. (2020). Developing CRISPR-based sex-ratio distorters for the genetic control of fruit fly pests: A how to manual. Arch. Insect Biochem. Physiol..

[20] Kandul N.P., Liu J., Sanchez C.H., Wu S.L., Marshall J.M., Akbari O.S. (2019). Reply to ‘Concerns about the feasibility of using “precision guided sterile males” to control insects’. Nat. Commun..

[21] Kandul N.P., Liu J., Sanchez C.H.M., Wu S.L., Marsha J.M., Akbari O.S. (2019). Transforming insect population control with precision guided sterile males with demonstration in flies. Nat. Commun..

[22] Gratz S.J., Cummings A.M., Nguyen J.N., Hamm D.C., Donohue L.K., Harrison M.M., Wildonger J., O’Connor-Giles K.M. (2013). Genome Engineering of *Drosophila* with the CRISPR RNA-Guided Cas9 Nuclease. Genetics.

[23] Cong L., Ran F.A., Cox D., Lin S.L., Barretto R., Habib N., Hsu P.D., Wu X.B., Jiang W.Y., Marraffini L.A. (2013). Multiplex genome engineering using CRISPR/Cas systems. Science.

[24] Aumann R.A., Schetelig M.F., Häcker I. (2018). Highly efficient genome editing by homology-directed repair using Cas9 protein in *Ceratitis capitata*. Insect Biochem. Mol. Biol..

[25] Ferguson L.C., Green J., Surridge A., Jiggins C.D. (2011). Evolution of the insect *yellow* gene family. Mol. Biol. Evol..

[26] Wittkopp P.J., Vaccaro K., Carroll S.B. (2002). Evolution of *yellow* gene regulation and pigmentation in *Drosophila*. Curr. Biol..

[27] Arnoult L., Su K.F.Y., Manoel D., Minervino C., Magrina J., Gompel N., Prud’homme B. (2013). Emergence and diversification of fly pigmentation through evolution of a gene regulatory module. Science.

[28] Hinaux H., Bachem K., Battistara M., Rossi M., Xin Y.Q., Jaenichen R., Le Poul Y., Arnoult L., Kobler J.M., Kadow I.C.G. (2018). Revisiting the developmental and cellular role of the pigmentation gene *yellow* in *Drosophila* using a tagged allele. Dev. Biol..

[29] Gokcezade J., Sienski G., Duchek P. (2014). Efficient CRISPR/Cas9 plasmids for rapid and versatile genome editing in *Drosophila*. G3.

[30] Kondo S., Ueda R. (2013). Highly improved gene targeting by germline-specific Cas9 expression in *Drosophila*. Genetics.

[31] Ren X.J., Sun J., Housden B.E., Hu Y.H., Roesel C., Lin S.L., Liu L.P., Yang Z.H., Mao D.C., Sun L.Z. (2013). Optimized gene editing technology for *Drosophila melanogaster* using germ line-specific Cas9. Proc. Natl. Acad. Sci. USA.

[32] Gratz S.J., Ukken F.P., Rubinstein C.D., Thiede G., Donohue L.K., Cummings A.M., O’Connor-Giles K.M. (2014). Highly specific and efficient CRISPR/Cas9-catalyzed homology-directed repair in *Drosophila*. Genetics.

[33] Xue Z.Y., Ren M.D., Wu M.H., Dai J.B., Rong Y.K.S., Gao G.J. (2014). Efficient gene knock-out and knock-in with transgenic Cas9 in *Drosophila*. G3.

[34] Port F., Chen H.M., Lee T., Bullock S.L. (2014). Optimized CRISPR/Cas tools for efficient germline and somatic genome engineering in *Drosophila*. Proc. Natl. Acad. Sci. USA.

[35] Akbari O.S., Oliver D., Eyer K., Pai C.Y. (2009). An Entry/Gateway (R) cloning system for general expression of genes with molecular tags in *Drosophila melanogaster*. BMC Cell Biol..

[36] Schwirz J., Yan Y., Franta Z., Schetelig M.F. (2020). Bicistronic expression and differential localization of proteins in insect cells and *Drosophila suzukii* using picornaviral 2A peptides. Insect Biochem. Mol. Biol..

[37] Kalajdzic P., Schetelig M.F. (2017). CRISPR/Cas-mediated gene editing using purified protein in *Drosophila suzukii*. Entomol. Exp. Appl..

[38] Yan Y., Ziemek J., Schetelig M.F. (2020). CRISPR/Cas9 mediated disruption of the *white* gene leads to pigmentation deficiency and copulation failure in *Drosophila suzukii*. J. Insect Physiol..

[39] Karageorgi M., Braecker L.B., Lebreton S., Minervino C., Cavey M., Siju K.P., Kadow I.C.G., Gompel N., Prud’homme B. (2017). Evolution of multiple sensory systems drives novel egg-laying behavior in the fruit pest *Drosophila suzukii*. Curr. Biol..

[40] Wittkopp P.J., Beldade P. (2009). Development and evolution of insect pigmentation: Genetic mechanisms and the potential consequences of pleiotropy. Semin. Cell Dev. Biol..

[41] Riedel F., Vorkel D., Eaton S. (2011). Megalin-dependent Yellow endocytosis restricts melanization in the Drosophila cuticle. Development.

[42] Wright T.R., Bewley G.C., Sherald A.F. (1976). The genetics of dopa decarboxylase in *Drosophila melanogaster*. II. Isolation and characterization of dopa-decarboxylase-deficient mutants and their relationship to the alpha-methyl-dopa-hypersensitive mutants. Genetics.

[43] Wright T.R., Hodgetts R.B., Sherald A.F. (1976). The genetics of dopa decarboxylase in *Drosophila melanogaster*. I. Isolation and characterization of deficiencies that delete the dopa-decarboxylase-dosage-sensitive region and the alpha-methyl-dopa-hypersensitive locus. Genetics.

[44] Sterkel M., Ons S., Oliveira P.L. (2019). DOPA decarboxylase is essential for cuticle tanning in *Rhodnius prolixus* (Hemiptera: *Reduviidae*), affecting ecdysis, survival and reproduction. Insect Biochem. Mol. Biol..

[45] Drapeau M.D., Radovic A., Wittkopp P.J., Long A.D. (2003). A gene necessary for normal male courtship, *yellow*, acts downstream of *fruitless* in the *Drosophila melanogaster* larval brain. J. Neurobiol..

[46] Cobb M. (2007). A gene mutation which changed animal behaviour: Margaret Bastock and the yellow fly. Anim. Behav..

[47] Sitaraman D., Zars M., LaFerriere H., Chen Y.C., Sable-Smith A., Kitamoto T., Rottinghaus G.E., Zars T. (2008). Serotonin is necessary for place memory in *Drosophila*. Proc. Natl. Acad. Sci. USA.

[48] Xiao C.F., Qiu S., Robertson M. (2017). The *white* gene controls copulation success in *Drosophila melanogaster*. Sci. Rep..

[49] Dow M.A. (1976). The genetic basis of receptivity of *yellow* mutant *Drosophila melanogaster* females. Behav. Genet..

[50] Pirrotta V., Steller H., Bozzetti M.P. (1985). Multiple upstream regulatory elements control the expression of the *Drosophila white* Gene. EMBO J..

[51] Lucchesi J.C., Kuroda M.I. (2015). Dosage Compensation in *Drosophila*. Cold Spring Harb. Perspect. Biol..

[52] Bassett A.R., Tibbit C., Ponting C.P., Liu J.L. (2013). Highly efficient targeted mutagenesis of *Drosophila* with the CRISPR/Cas9 system. Cell Rep..

[53] Massey J.H., Chung D., Siwanowicz I., Stern D.L., Wittkopp P.J. (2019). The yellow gene influences *Drosophila* male mating success through sex comb melanization. eLife.

[54] Meltzer H., Marom E., Alyagor I., Mayseless O., Berkun V., Segal-Gilboa N., Unger T., Luginbuhl D., Schuldiner O. (2019). Tissue-specific (ts) CRISPR as an efficient strategy for in vivo screening in *Drosophila*. Nat. Commun..

[55] Port F., Strein C., Stricker M., Rauscher B., Heigwer F., Zhvou J., Beyersdorffer C., Frei J., Hess A., Kern K. (2020). A large-scale resource for tissue-specific CRISPR mutagenesis in *Drosophila*. Elife.

[56] Jaffri S.A., Yan Y., Scott M.J., Schetelig M.F., Garcia F.R.M. (2021). Conditional expression systems for *Drosophila suzukii* pest control. Drosophila Suzukii Management.

[57] Meccariello A., Krsticevic F., Colonna R., Del Corsano G., Fasulo B., Papathanos P.A., Windbichler N. (2021). Engineered sex ratio distortion by X-shredding in the global agricultural pest *Ceratitis capitata*. BMC Biol..

[58] Champer J., Chung J., Lee Y.L., Liu C., Yang E., Wen Z.X., Clark A.G., Messer P.W. (2019). Molecular safeguarding of CRISPR gene drive experiments. Elife.

[59] Akbari O.S., Bellen H.J., Bier E., Bullock S.L., Burt A., Church G.M., Cook K.R., Duchek P., Edwards O.R., Esvelt K.M. (2015). Safeguarding gene drive experiments in the laboratory. Science.

[60] Schetelig M.F., Handler A.M. (2012). Strategy for enhanced transgenic strain development for embryonic conditional lethality in *Anastrepha suspensa*. Proc. Natl. Acad. Sci. USA.

[61] Doench J.G., Hartenian E., Graham D.B., Tothova Z., Hegde M., Smith I., Sullender M., Ebert B.L., Xavier R.J., Root D.E. (2014). Rational design of highly active sgRNAs for CRISPR-Cas9-mediated gene inactivation. Nat. Biotechnol..

[62] Hsu P.D., Scott D.A., Weinstein J.A., Ran F.A., Konermann S., Agarwala V., Li Y.Q., Fine E.J., Wu X.B., Shalem O. (2013). DNA targeting specificity of RNA-guided Cas9 nucleases. Nat. Biotechnol..

