# Supplementary material for: Highly Efficient Temperature Inducible CRISPR-Cas9 Gene Targeting in Drosophila suzukii
Source: Int J Mol Sci. 2021 Jun 23;22(13):6724. doi: 10.3390/ijms22136724 (PMC8268499; doi:10.3390/ijms22136724)
Supplement: Supplementary file 1 [file ijms-22-06724-s001.zip › ijms-1255872-supplementary.pdf]

Highly Efficient Temperature Inducible CRISPR-Cas9 Gene Targeting in *Drosophila suzukii*

Ying Yan [1,*], Yukino Kobayashi [1], Cong Huang [2,3], Bo Liu [2,3], Wanqiang Qian [2,3], Fanghao Wan [2,3] and Marc F. Schetelig [1]

1 Department of Insect Biotechnology in Plant Protection, Institute for Insect Biotechnology, Justus-Liebig-University Giessen, Winchesterstraße 2, 35394 Giessen, Germany; Yukino.Kobayashi@ernaehrung.uni-giessen.de (Y.K.); Marc.Schetelig@agrar.uni-giessen.de (M.F.S.)

2 Shenzhen Branch, Guangdong Laboratory of Lingnan Modern Agriculture, Agricultural Genomics Institute at Shenzhen, Chinese Academy of Agricultural Sciences, Shenzhen 518120, China; huangcong16@163.com (C.H.); liubo03@caas.cn (B.L.); qianwanqiang@caas.cn (W.Q.); wanfanghao@caas.cn (F.W.)

3 Genome Analysis Laboratory of the Ministry of Agriculture and Rural Affairs, Agricultural Genomics Institute at Shenzhen, Chinese Academy of Agricultural Sciences, Shenzhen 518120, China; huangcong16@163.com (C.H.); liubo03@caas.cn (B.L.); qianwanqiang@caas.cn (W.Q.); wanfanghao@caas.cn (F.W.)

* Correspondence: Ying.Yan@agrar.uni-giessen.de

Supplemental tables S1–4 and figures S1–4.

Table S1. Primer sequences

Primer No.	Primer Name	Primer Sequence
P212	Bsp119I_attP_F	ACTTTCGAAACTAGTACTGACGGAC
P213	Bsp119I_attP_R	TCATTCGAACTGTACTAGTCGCGCTC
MFS10	pBac_5'_R	ACGACCGCGTGAGTCAAAATGACG
MFS17	EGFP_3'_F	CAGAACACCCCCATCGGCGACGGC
P109	SV40_3'_F	CAGCCATACCACATTTGTAGAGG
P106	EGFP_RT-F	CAAAGACCCCAACGAGAAGC
P108	EGFP_RT-R	GTCCATGCCGAGAGTGATCC
P1489	Cas9_RT-F	GCCACCGTGCGGAAAGTGCTGAGC
P1490	Cas9_RT-R	CGGTGGGGCTGTCGAAGCCGCCG
P740	DsTBP_RT-F	CCACGGTGAATCTGTGCT
P741	DsTBP_RT-R	GGAGTCGTCCTCGCTCTT
P898	DsY_ORF-F	ATGTTTCAGGACAAAGGATGGATCCTGGTGA
P899	DsY_ORF-R	TTAACCCTGATGCTGGTGGTGCCACCAGCCAGA
P145	EGFP_genotype_F	ACTTAATCGCCTTGCAGCACATCC
P55	EGFP_genotype_R	TGTGATCGCGCTTCTCGTT
P1720	DsY_genotype_F	CTGACTGGTTCGCCGGAGCTG
P1808	DsY_genotype_R	CCAGCCACATTGAAGTCACCCCTC
P1172	sgRNA_EGFP2b-F	GAAATTAATACGACTCACTATAGGCTGAAGCACTGCACGCCGTGTTT TAGAGCTAGAAATAGC
P1686	sgRNA_Ds_Y_E2-F	GAAATTAATACGACTCACTATAGGTCGACCTGACCACCGATACGGTT TTAGAGCTAGAAATAGC
P369	sgRNA_UNI-R	AAAAGCACCGACTCGGTGCCACTTTTTCAAGTTGATAACGGACTAGC CTTATTTTAACTTGCTATTTCTAGCTCTAAAAC

Table S2. Germline transmission of EGFP mutations under different heat-shock conditions

Injection mix (HS condition)[a]	Founder G_0[b]	G_1 mutants	Total No. of G_1	Inheritance from G_0 to G_1[c]	Average inheritance	No. of detected mutations[d]
V23 plasmid (Non-HS)	F18	42	74	42/74 (56.8%)	56.8%	1
EGFP-2b (Non-HS)	M2	154	315	154/315 (48.9%)	39.4%	2
	M6	82	313	82/313 (26.2%)		1
	M7	84	85	84/85 (98.8%)		1
	F4	13	143	13/143 (9.1%)		1
	F5	6	43	6/43 (14.0%)		1
EGFP-2b (HS-after 20 h)	M2	83	125	83/125 (66.4%)	78.3%	2
	M3	7	195	7/195 (3.6%)		1
	F2	8	8	8/8 (100%)		2
	F3	83	83	83/83 (100%)		2
	F5	25	25	25/25 (100%)		1
	F9	15	15	15/15 (100%)		2
EGFP-2b (HS-parents)	M2	193	203	193/203 (95.1%)	92.0%	2
	M4	169	180	169/180 (89.0%)		1

[a] The composition of the injection mix and the heat-shock (HS) conditions are the same as in Table. 1

[b] The G_0 flies that transmitted Cas9-derived mutant alleles to the G_1 generation.

[c] The number of mutant G_1 offspring relative to the total number of G_1 offspring.

[d] The number of verified mutations in the target EGFP region from 2-5 mutant (non-fluorescent) G_1 flies from each G_0 cross.

Table S3. Injection and crossing scheme to obtain *D. suzukii yellow* mutants (y^-)

Injection mix (HS condition)[a]	Injected embryos	Hatched larvae	Mosaic G_0 adults[b]	G_0 group crosses[c]	Ratio of y^- males in G_1[d]	Ratio of $y^{-/+}$ females in G_1[d]	G_0 founders[e]
Ds_y_E2 (HS-after 20 h)	435	50	5.3% (2/38)	M_{1-5}	2.6% (14/536)	1.9% (10/536)	2.6% (1/38)
				M_{6-10}	0	0	
				M_{11-15}	0	0	
				M_{16-20}	0	0	
				M_{20-25}	0	0	
				M_{26-27}	0	0	
				F_{1-11}	0	0	

[a] The DsY_E2 is the synthesized gRNA (200 ng/µL) that targeting the second exon (E2) of *DsY* gene, and the heat-shock (HS) condition is the same as in Table. 1.

[b] The number of mosaic individuals relative to the total number of G_0 adults.

[c] The G_0 males or females were group crossed to wildtype (WT) partners (15 WT females were used for each G_0 male group and 11 WT males were used for the G_0 female group).

[d] The number of *yellow* mutant flies relative to the total number of G_1 offspring.

[e] The number of G_0 founder flies that predicted by the number of mutation events relative to the number of G_0 adults.

Table S4. Lethality tests for *DsY*$^{-}$ males over multiple generations

Flies	Generation (G)[a]	No. of newly emerged flies[b]	No. of flies after 1 day	No. of flies after 2 day	No. of flies after 10 day
DsY$^{-/+}$ female	G2	7	7	7	7
DsY^{-} male	G2	15	5	0	
	G3	15	2	0	
	G4	15	1	0	
	G5	15	3	0	
	G6	15	1	0	
	G7	15	1	0	
	G8	15	2	0	
	G9	15	1	0	
	G10	15	2	0	
	G11	15	3	0	
	G12	15	1	0	
	G13	15	1	0	
	G14	15	1	0	
	G15	15	2	0	
	G16	15	1	0	
	G17	15	1	0	
	G18	15	1	0	
	G19	15	3	0	
	G20	15	2	0	
DsY$^{-/+}$ female	G20	15	15	14	13
WT male		15	15	15	11

[a] The *yellow* mutant heterozygous females (*DsY*$^{-/+}$) were crossed with wildtype (WT) males in each generation for population maintenance due to the lethality in *yellow* mutant males (*DsY*$^{-}$).

[b] The newly emerged flies (<4 hours) were placed into fresh food vials, and the number (No.) of flies were counted every day until day 10 or all flies died.

sgRNA-EGFP1

Target Sequence: CTCGCCCTTGCTCACCATGG

Protospacer Adjacent Motif (PAM): TGG

Doench (2014) Activity Score: 0.027

Zhang (2013) Specificity Score: 99.40%

Off-target Sites: 3 (0 in CDS)

Off-target #1 Score: 1.00%
Sequence (#1): CTCGTCCTTGCCCGCCATGGTGG

Off-target #2 Score: 1.00%
Sequence (#2): CTCGTCCTTGCCCGCCATGGTGG

Off-target #3 Score: 1.00%
Sequence (#3): CTCGTCCTTGCCCGCCATGGTGG

sgRNA-EGFP2b

Target Sequence: GCTGAAGCACTGCACGCCGT

Protospacer Adjacent Motif (PAM): AGG

Doench (2014) Activity Score: 0.329

Zhang (2013) Specificity Score: 100.00%

Off-target Sites: 0 (0 in CDS)

sgRNA-EGFP2

Target Sequence: GTCGCCACCATGGTGAGCAA

Protospacer Adjacent Motif (PAM): GGG

Doench (2014) Activity Score: 0.701

Zhang (2013) Specificity Score: 100.00%

Off-target Sites: 0 (0 in CDS)

sgRNA-Ds_y_E2

Target Sequence: TCGACCTGACCACCGATACG

Protospacer Adjacent Motif (PAM): AGG

Doench (2014) Activity Score: 0.684

Zhang (2013) Specificity Score: 100.00%

Off-target Sites: 0 (0 in CDS)

Figure 1. Off-target analysis for the gRNAs. Assessment of potential off-target effects of each gRNA was performed using the "Find CRISPR sites" tool of the Geneious Software Package 11.0.6 (Kearse et al., 2012) based on the *D. suzukii* genome (Dsuzukii_v1_scaffolds.fa.gz) (Chiu et al., 2013). The activity of gRNA was predicted through its sequence features based on the model proposed by Doench et al. (2014), and scores are between 0 and 1, with a higher score denoting higher expected activity. The specificity of gRNA was scored based on the Zhang (2013) method by searching for CRISPR sites with 3' PAMs, and scores are between 0 and 100, with a higher score meaning better specificity and less off-target activity. The numbers of the potential off-target sequences in the target genome and coding region sequence (CDS) were indicated. Each off-target sequence was given a score based on how similar it was to the spacer sequence of gRNAs. A higher score (%) indicated a higher similarity to the original target site and a higher likelihood to cause off-target mutations.

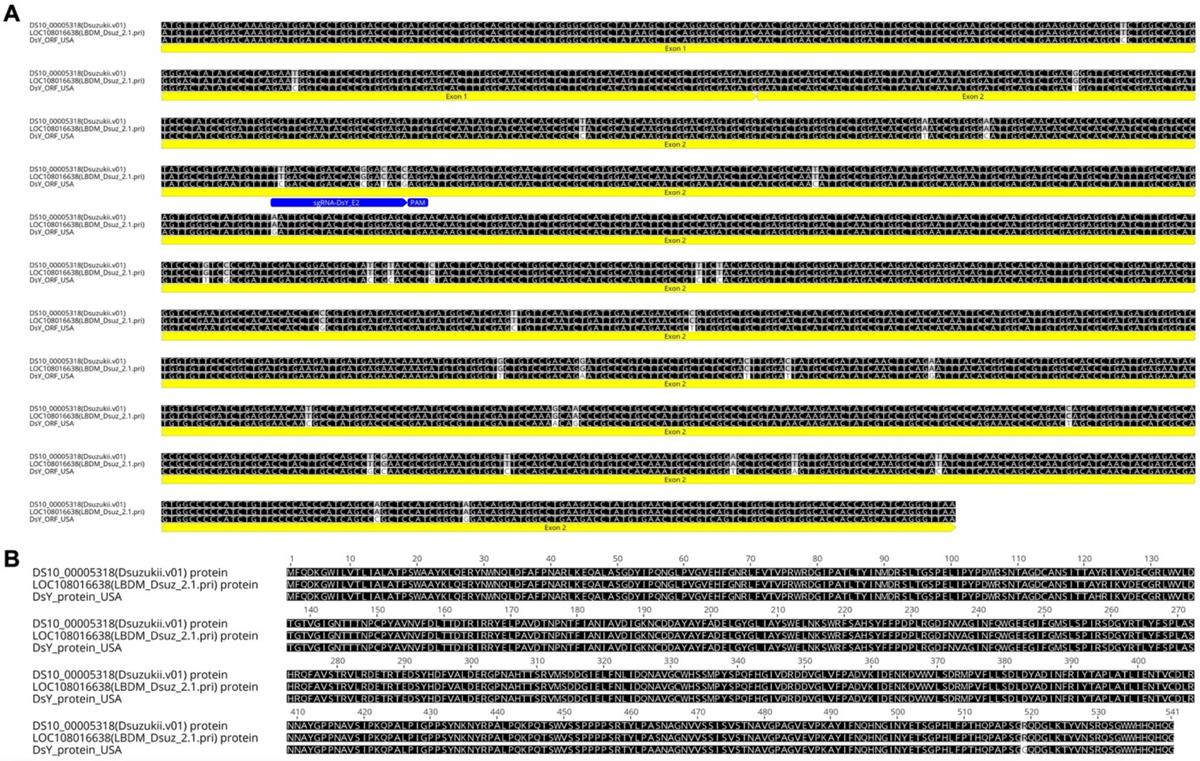

Figure S2. Multiple sequence alignment of *D. suzukii yellow* alleles (A) and proteins (B). DS10_00005318 is from a previously assembled reference genome (Dsuzukii.v01) (Chiu et al. 2013). LOC108016638 is from a recently assembled genome (LBDM_Dsuz_2.1.pri) (Paris et al., 2020). DsY_ORF_USA is the open reading frame that was isolated from a laboratory wildtype (USA) strain. sgRNA and PAM sequences are indicated in blue boxes. The protein sequences were predicted from the isolated and in silico *yellow* sequences using the Geneious Prime software.

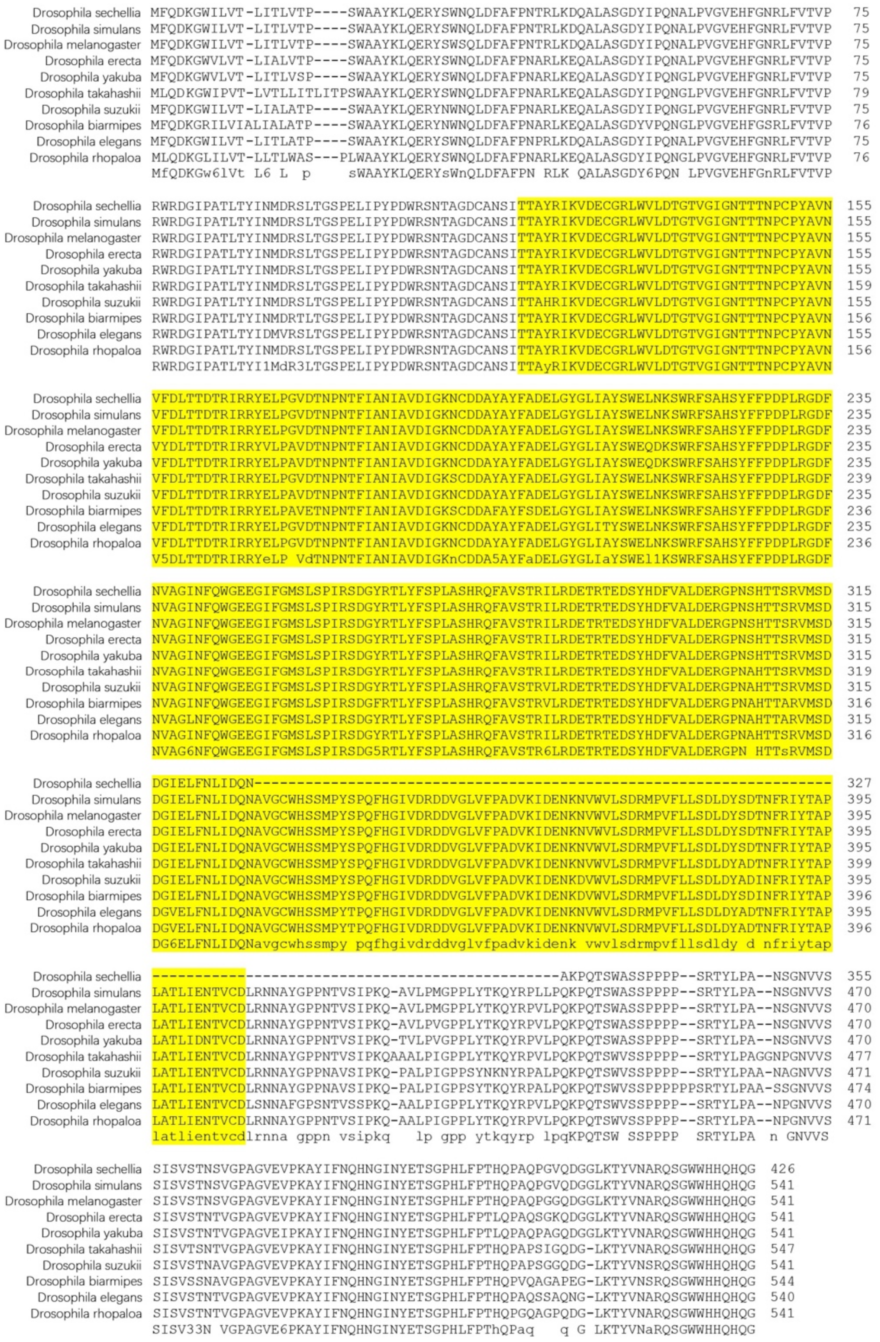

Figure S3. Multiple sequence alignment of Yellow from ten *Drosophila* species. The alignment was performed by MAFFT v7. Aligned sequences were depicted with ESPript 3.0 server (http://espript.ibcp.fr/ESPript/cgi-bin/ESPript.cgi). The conserved Major Royal Jelly Protein (MRJP; pfam03022) domain is highlighted in yellow. Dsec: *Drosophila sechellia* (XP_002040222.1), Dsim: *Drosophila simulans* (XP_016037524.1), Dmel: *Drosophila melanogaster* (NP_476792.1), Dere: *Drosophila erecta* (XP_001982384.1), Dyak: *Drosophila yakuba* (XP_002099667.1), Dtak: *Drosophila takahashii* (XP_016995421.1), Dsuz: *Drosophila suzukii* (this study), Dbia: *Drosophila biarmipes* (XP_016948849.1), Dele: *Drosophila elegans* (XP_017114065.1), Drho: *Drosophila rhopaloa* (XP_016982272.1).

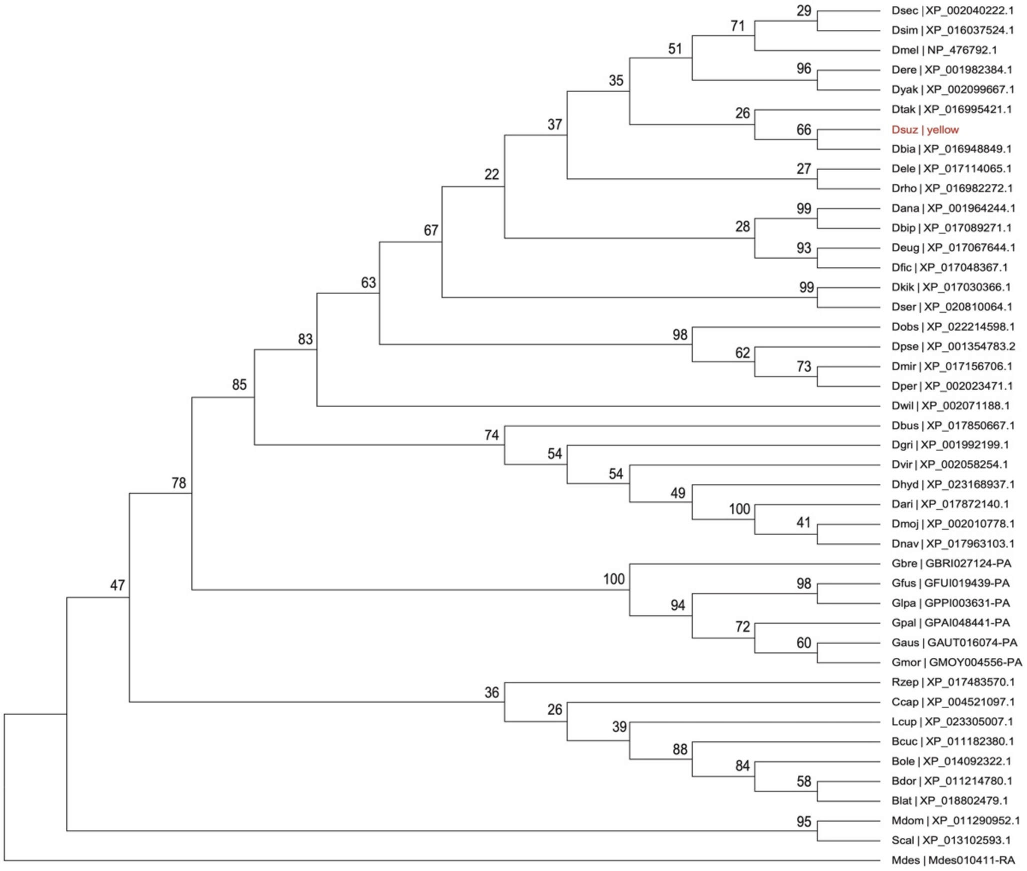

Figure S4. Phylogenetic tree of Yellow from 44 insect species. The neighbor-joining phylogenetic tree was constructed using the aligned sequences by the ClustalW algorithm in MEGA6 with the JTT substitution model. A bootstrap method of 1000 replicates was used to evaluate the branch strength of the model. The *D. suzukii* Yellow from this study is indicated in red, and the GenBank accession numbers for Yellow from other species are shown. Dsec: *Drosophila sechellia*, Dsim: *Drosophila simulans*, Dmel: *Drosophila melanogaster*, Dere: *Drosophila erecta*, Dyak: *Drosophila yakuba*, Dtak: *Drosophila takahashii*, Dsuz: *Drosophila suzukii*, Dbia: *Drosophila biarmipes*, Dele: *Drosophila elegans*, Drho: *Drosophila rhopaloa*, Dana: *Drosophila ananassae*, Dbip: *Drosophila bipectinata*, Deug: *Drosophila eugracilis*, Dfic: *Drosophila ficusphila*, Dkik: *Drosophila kikkawai*, Dser: *Drosophila serrata*, Dobs: *Drosophila obscura*, Dpse: *Drosophila pseudoobscura*, Dmir: *Drosophila miranda*, Dper: *Drosophila persimilis*, Dwill: *Drosophila willistoni*, Dbus: *Drosophila busckii*, Dgri: *Drosophila grimshawi*, Dvir: *Drosophila virilis*, Dhyd: *Drosophila hydei*, Dari: *Drosophila arizonae*, Dmoj: *Drosophila mojavensis*, Dnav: *Drosophila navojoa*, Gbre: *Glossina brevipalpis*, Gfus: *Glossina fuscipes*, Glpa: *Glossina palpalis*, Gpal: *Glossina pallidipes*, Gaus: *Glossina austeni*, Gmor: *Glossina morsitans*, Rzep: *Rhagoletis zephyria*, Ccap: *Ceratitis capitata*, Lcup: *Lucilia cuprina*, Bcuc: *Bactrocera cucurbitae*, Bole: *Bactrocera oleae*, Bdor: *Bactrocera dorsalis*, Blat: *Bactrocera latifrons*, Mdom: *Musca domestica*, Scal: *Stomoxys calcitrans*, Mdes: *Mayetiola destructor*.
